# Preventable Emergency Department Utilization Among Patients With Foster Care History Compared to Patients Without Foster Care History

**DOI:** 10.1177/10775595241300971

**Published:** 2024-11-25

**Authors:** Lauren Q. Malthaner, Jill D. McLeigh, Gregory Knell, Katelyn K. Jetelina, Folefac Atem, Sarah E. Messiah

**Affiliations:** 1 Department of Epidemiology, 12340 UTHealth Houston School of Public Health, Dallas, TX, USA; 2 Center for Pediatric Population Health, 12340 UTHealth Houston School of Public Health, Dallas, TX, USA; 3 Rees-Jones Center for Foster Care Excellence, 2755 Children’s Health Medical Center, Dallas, TX, USA; 4 12376 The University of North Texas Health Science Center School of Public Health, Fort Worth, TX, USA; 5 0.05 Findings LLC, San Diego, CA, USA; 6 Department of Biostatistics, 12340 UTHealth Houston School of Public Health, Dallas, TX, USA; 7 Department of Pediatrics, McGovern Medical School, Houston, TX, USA

**Keywords:** pediatric emergency department, emergency department utilization, health care utilization, foster care, foster children

## Abstract

Emergency department (ED) utilization for preventable reasons by patients with foster care history is unexplored. Medical records of ED encounters from primary care patients were pulled from a southwestern children’s hospital system. Necessity of ED encounter was categorized using the New York University- ED Algorithm into emergent, intermediate, or non-emergent. Associations were explored at the encounter- and patient-level. Partial proportional logistic models generated odds of preventable (i.e., intermediate or nonemergent) ED utilization among encounters, and Poisson models determined incidence of preventable ED use at the patient level. Findings suggested that when a patient with history in foster care used the ED, the odds that it was preventable were lower than if the child did not have such experience. Further, patients with foster care history were less likely to use the ED for concerns that did not need immediate attention but were more likely to use the ED for intermediate reasons.

## Introduction

Preventable emergency department (ED) encounters are those where a patient was seen in the ED when either ED intervention was not needed or could have been avoided with preventative care. Such encounters place a large burden on the U.S. healthcare system. Preventable ED utilization costs the U.S. healthcare system an estimated $83 billion per year (Daly, 2019). One study found that among patients with encounters at an urban pediatric health system, patients in foster care had higher healthcare charges per 100 patient-years compared to those not in foster care (foster care: $82,886 [95% CI: 77.635–98,137]; non-foster care: $40,656 [95% CI: 47–126]) (Bennett et al., 2020). Patients in foster care have high proportions of special healthcare needs, thereby putting them at high risk for using the ED for preventable encounters, resulting in increased healthcare costs (*Children and Youth with Special Health Care Needs (CYSHCN)*, 2022; *Mental and Behavioral Health Needs of Children in Foster Care*, 2021).

Limited research, however, has examined ED use by patients in foster care, and results are mixed among the few studies that do exist. One study showed patients who had Child Protective Services reports, a precursor to entering the foster care system, had greater odds of using the ED (OR: 1.9; 95% CI: 1.4–2.5) than patients without Child Protective Services reports (Carr et al., 2020). A second study showed a nonsignificant increase in rates of ED encounters for patients in foster care compared to non-foster care patients (Incidence rate ratio [IRR]: 1.2 [95% CI: 1.0–1.3]) (Bennett et al., 2020). Additionally, among patients in foster care, it has been shown that younger patients (0–2 years: Reference; 3–5 years OR: 0.51 [95% CI: 0.19–1.33], 6–10 years OR: 0.26 [95% CI: 0.13–0.53], ≥11 years OR: 0.37 [95% CI: 0.22–0.61]) and patients with chronic conditions (OR: 1.96 [95% CI: 1.17–3.27]) utilize the ED significantly more than their pediatric counterparts, respectively (Jee et al., 2005). Patients in foster care have also been shown to use the ED frequently for injuries (32.2% of ED encounters), especially those related to intentional injury (44.1% of injury ED encounters) (Thackeray et al., 2016). Therefore, a better understanding of the healthcare utilization patterns of patients in foster care has significant implications for future care planning and intervention and, in the case of ED encoutners, a potential decrease in healthcare-related costs. More specifically, detailed knowledge regarding overall ED use, including appropriateness of use (e.g., for urgent, high-acuity complaints) among pediatric subpopulations and identifying populations with higher levels of ED misuse can help identify possible intervention targets.

Andersen’s behavioral model of health care services use is a theoretical framework that has been used to explore factors that impact the decision to use the ED for preventable reasons (Uscher-Pines et al., 2013). This model identifies three core components that help determine this usage: (1) predisposing factors; (2) enabling factors; and (3) need factors (Andersen, 1968). Predisposing factors include social factors which encompass family status. Children in foster care have unstable family situations related to their involvement with the child welfare system. Enabling factors include the ability to pay for health services. The large majority of children in foster care in the U.S. are eligible for Medicaid insurance as a result of their placement, thus the financial situation enables them to utilize more healthcare services (Child Welfare Information Gateway, 2022). Finally, need factors include patient’s health status. As stated, children in foster care have high proportions of special healthcare needs which may influence their perceived need for services (*Children and Youth with Special Health Care Needs (CYSHCN)*, 2022; *Mental and Behavioral Health Needs of Children in Foster Care*, 2021). Given that this population has at least one predisposing factor, enabling factor, and need factor, it is plausible that decision-making processes lead this group to utilize the ED more frequently for preventable reasons.

The primary objectives of this study were to (a) assess preventable ED utilization among patients with foster care experience relative to those without foster care experience and, (b) explore demographic characteristics of patients that are associated with preventable ED usage. We hypothesized that patients with foster care experience would be more likely to use the ED for preventable reasons at both the patient and encounter (i.e., visit) level. We further hypothesized that the relationship between various demographic factors and preventable ED use would be similar to those found in studies looking at ED utilization in general populations and in foster care pediatric populations. Specifically, we hypothesized that younger age, female gender, Hispanic ethnicity, and more complex health status would be associated with greater utilization of the ED for preventable reasons.

## Methods

### Data Source

Data for this cross-sectional study were collected from the patient electronic medical record of patients seen at the ED at the Children’s Medical Center of Dallas, Dallas and Plano campuses who were also primary care patients seen at either the Dallas or Plano campuses of the Rees-Jones Center for Foster Care Excellence or Dallas Medical Group Primary Care, the primary care clinic at Children’s Medical Center of Dallas. All eligible encounters of patients who were seen in the ED between January 1, 2018 and December 31, 2021 were included in the analysis. Analyses for this study were conducted on two levels of interest: patient-level and encounter-level. All study procedures were approved by the Institutional Review Board at University of Texas Southwestern Medical Center and UTHealth Houston School of Public Health.

### Measures

The primary outcome was whether an ED encounter was preventable based on two levels of analysis: encounter-level, using the single visit as the unit of analysis, and patient-level, using the patient as the unit of analysis. Both units of analysis are included given their different implications for the understanding of healthcare utilization as well as intervention planning. Encounter-level analyses are helpful to describe general ED utilization and understand care requirements for the populations of interest (Fertel et al., 2012). In this study, it can help explore the likelihood of a child with a history of foster care requiring emergency resources at time of presentation to the ED compared to those never in foster care. A patient-level analysis, on the other hand, helps determine if these results are impacted by repeated encounters among the same patients, which can help determine if this population are frequent ED utilizers for preventable reasons and thus could potentially benefit from interventions related to decreasing frequent encounters (Fertel et al., 2012).

**Figure 1.**
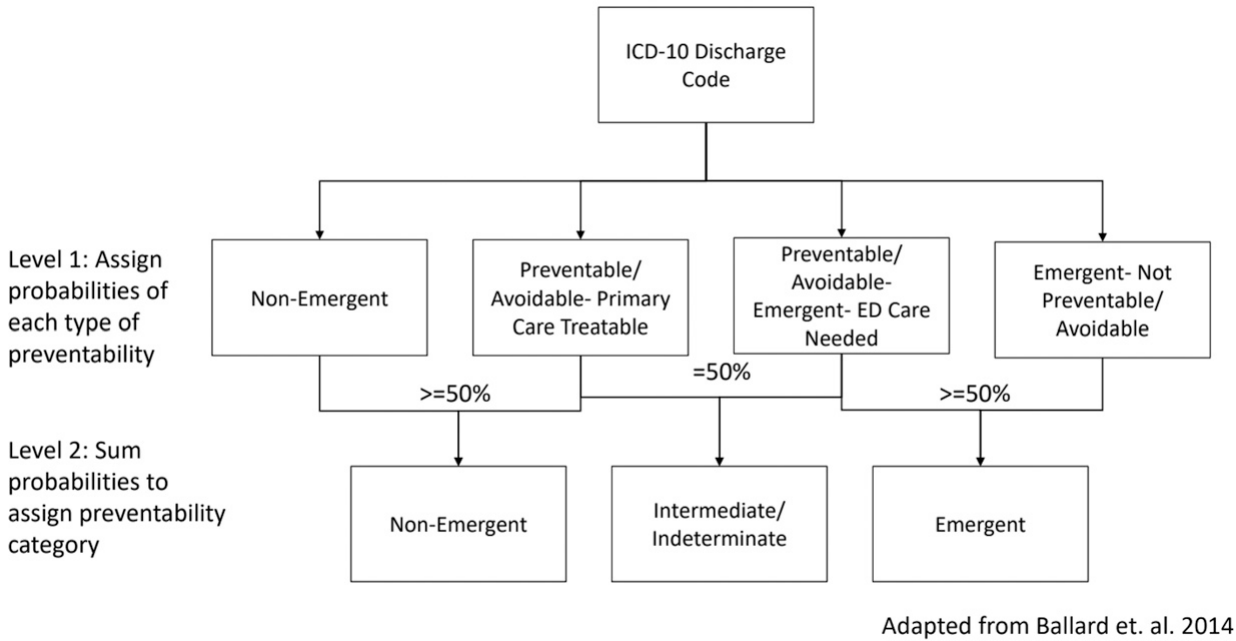
Categorization process of New York University- emergency department algorithm.

The New York University Emergency Department (NYU-ED) algorithm, an algorithm that has been used nationally to determine severity of misuse of ED resources among populations, was used to categorize preventable ED utilization using a two-level categorization (Figure 1, Billings et al., 2000; Johnston et al., 2017). First, the NYU-ED Algorithm categorizes the probability of the primary discharge ICD-10 code for each encounter fitting into four categories: Non-Emergent (i.e., immediate medical care was not required within 12 hours), Emergent/Primary Care Treatable (i.e., treatment was required withing 12 hours but could have been administered in a primary care setting), Emergent- ED Care Needed- Preventable/Avoidable (i.e., emergency department treatment was needed but was preventable if ambulatory care had been appropriately received), and Emergent- ED Care Needed- Not Preventable/Avoidable (i.e., emergency care was required and not preventable). Secondly, the NYU-ED Algorithm further classifies these encounters into three categories: Non-emergent, Intermediate and Emergent. Consistent with other studies, encounters were classified as emergent if the sum of the probabilities of Emergent- ED Care Needed- Not Preventable/Avoidable and Emergent- ED Care Needed- Preventable/Avoidable were greater than 50% (Ballard et al., 2010; Gandhi & Sabik, 2014). Encounters were classified as non-emergent if the sum of the probabilities of Non-Emergent and Emergent-Primary Care Treatable were greater than 50%. If either of these sums equated to exactly 50%, they were categorized as intermediate (i.e., the encounter had equal probability of being both non-emergent and emergent). In our study, preventable encounters were non-emergent and/or intermediate while emergent encounters served as the reference group (Ballard et al., 2010; Gandhi & Sabik, 2014). No injury, mental health, alcohol- or substance abuse-related codes are categorized in the algorithm. The encounter level analysis outcome was an ordinal variable indicating level of preventability (0 = emergent [Reference], 1 = intermediate, 2 = non-emergent). Three separate variables indicating the number of non-emergent encounters, the number of intermediate encounters, and the number of emergent encounters were created to define the person-level dependent outcome.

The primary exposure was history of foster care during the study period (predisposing factor; 0 = never in foster care [Reference], 1 = ever in foster care), which was measured using insurance status and foster clinic status. If patients had Star Health insurance or if they were seen at the Rees-Jones Center for Foster Care Excellence during the study period, they were coded as ever in foster care. All children in foster care in the State of Texas are provided with Star Health insurance, a Medicaid managed-care insurance program (*About Star Health*, n.d). To be eligible for Star Health insurance, patients must be in foster care or have aged out of foster care and are under voluntary foster care agreements or are eligible for Medicaid services (Supreme Court of Texas Permanent Judicial Commission for Children, Youth and Families, 2013). STAR Health provides full coverage for services covered under the insurance to all members (Superior Healthplan, 2023).

Covariates included in this analysis were Age at encounter/Age at first encounter, defined using the National Institute of Child Health and Development age categories (predisposing factor; 0 = 0 year [Reference], 1 = 1 year, 2 = 2–5 years, 3 = 6–11 years, 4 = 12–18 years), sex (predisposing factor; 0 = Male [Reference], 1 = Female, 2 = Unknown), race/ethnicity (predisposing factor; 0 = Non-Hispanic White [NHW, Reference], 1 = Non-Hispanic Black [NHB], 2 = Hispanic, 3 = Non-Hispanic Other, 4 = Unknown), and medical complexity (need factor). The Pediatric Medical Complexity Algorithm (PMCA) determined the chronicity and complexity of health conditions of patients seen in the ED (Simon et al., 2017). The PMCA categorizes medical conditions present as ICD-10 codes in the medical record into three categories: Non-Chronic, Non-complex Chronic, and Complex Chronic. Non-chronic conditions served as the reference category. There are three versions of the PMCA: least conservative, more conservative, and most conservative. For this study, the more conservative version was used because it has the most consistent sensitivity and specificity measurements, when assessed using a sample of Medicaid participants. The PMCA has a sensitivity of 87% and specificity of 91% for non-chronic classification, a sensitivity of 60% and specificity of 84% for non-complex chronic, and a sensitivity of 74% and specificity of 84% for complex chronic conditions (Simon et al., 2017).

### Statistical Analysis

Descriptive statistics, including means and standard deviations, and frequencies and percentages, were conducted. Bivariate analyses between history of foster care and age at first encounter, sex, race/ethnicity, medical complexity, time in dataset and urgency of encounter were conducted using chi-square tests of independence. Bivariate analyses between history of foster care and number of nonurgent ED encounters, number of intermediate ED encounters, and number of emergent ED encounters were conducted using Student’s t-tests. First, a two-level, mixed-effects partial proportional odds regression model clustered on patient tested the association between foster care status and preventable ED use for the encounter-level analysis. A *p*-value threshold of .05 was used to indicate statistical significance.

Secondly, negative binomial regression models calculated the incidence rate ratios of counts of non-emergent and emergent encounters, while a Poisson model calculated the incidence rate ratio of count of intermediate encounters for the patient level analysis. The best model fit was determined using Bayesian Information Criteria. Zero-inflation was not a concern as all patients included in the dataset had at least one valid ED encounter. Bonferroni adjustments for multiple comparisons were used for the patient-level analysis due to the three separate outcomes of interest, and a *p*-value of .017 based on dividing the standard .05 cutoff by the three outcome variables was used to indicate significance. All data management and analysis was conducted using Stata version 16.1.

## Results

### Encounter-level Analysis

There were 14,556 total encounters included in the sample with 18.8% of encounters being with patients ever in foster care (Table 1). Sex was majority male (51.8%), and most encounters were NHW (50.5%). NHB was the second most common race (38.9%). For encounters, 1.6% of the sample was Hispanic. Age at the encounter was primarily between 2 and 5 years of age (32.7%). Most encounters had non-chronic medical complexity (64.0%). Of encounters, 76.2% were non-emergent, while 20.2% were emergent.

**Table 1.** Descriptive and Bivariate Associations Between Demographic Characteristics and Encounter-level ED Necessity and Foster Care Status.

	All Encounters *n* = 14.556	Ever in Foster Care *n* = 2743	Never in Foster Care *n* = 11,813	*p*-Value
Age at encounter				
0 year	2010 (13.9)	581 (21.3)	1429 (12.2)	<0.001
1 year	2006 (13.8)	493 (18.1)	1513 (12.9)	
2–5 years	4736 (32.7)	869 (31.8)	3867 (32.9)	
6–11 years	3758 (25.9)	481 (17.6)	3277 (27.9)	
12–18 years	1983 (13.7)	307 (11.2)	1676 (14.3)	
Sex				
Male	7541 (51.8)	1416 (51.9)	6106 (51.9)	0.952
Female	7015 (48.2)	1315 (48.2)	5656 (48.1)	
Race/ethnicity				
Non-Hispanic white	7347 (50.5)	1249 (45.7)	6064 (51.6)	<0.001
Non-Hispanic black	5660 (38.9)	1235 (45.2)	4400 (37.4)	
Hispanic	238 (1.6)	9 (0.3)	227 (1.9)	
Other	1171 (8.0)	139 (5.1)	1030 (8.8)	
Unknown	140 (1.0)	99 (3.6)	41 (0.4)	
Medical complexity^a^				
Non-chronic	9275 (64.0)	1924 (70.5)	7451 (62.5)	<0.001
Non-complex chronic	3477 (24.0)	514 (18.8)	2963 (25.2)	
Complex chronic	1741 (12.0)	293 (10.7)	1448 (12.3)	
Urgency of encounter^b^				
Non-emergent	11,037 (76.2)	2008 (73.5)	9029 (76.8)	<0.001
Intermediate	628 (3.6)	183 (6.7)	345 (2.9)	
Emergent	2928 (20.2)	540 (19.7)	2388 (20.3)	

^a^Defined using the Pediatric Medical Complexity Algorithm.

^b^Defined using the NYU-Emergency Department Algorithm.

Table 1 also displays bivariate results of the encounter-level analysis. One in 5 (21.3%) encounters involving patients with a foster care history occurred in patients under 1 year of age. The majority of encounters for patients were NHW (45.7% history of foster care; 51.6% never in foster care), non-chronic medical complexity (70.5% history of foster care; 62.5% never in foster care), and were for non-emergent diagnoses (73.5% history of foster care; 62.5% never in foster care) (*p* < .001 for all).

Patients ever in foster care were less likely to use the ED for intermediate and non-emergent diagnoses than patients never in foster care when comparing intermediate and non-emergent conditions to emergent conditions (Odds Ratio [OR]: 0.87, 95% Confidence Interval [95% CI]: 0.77–0.99, Table 2) and for non-emergent diagnoses when comparing non-emergent diagnoses to intermediate and emergent diagnoses (OR: 0.74, 95% CI: 0.65–0.83). All age groups were significantly associated with decreased odds of using the ED for less urgent diagnoses compared to those less than one year of age. Specifically, patients who were one year old had 0.82 (95% CI: 0.69–0.97) times the odds of using the ED for less emergent diagnoses than patients less than one year. When comparing intermediate and non-emergent diagnoses to emergent diagnoses, patients 2–5 years of age, patients 6–11 years of age, and patients 12–18 years of age were less likely to use the ED for non-emergent complaints (OR: 0.74, 95% CI: 0.63–0.87; OR: 0.62, 95% CI: 0.52–0.73; OR: 0.57, 95% CI 0.47–0.69, respectively) than patients less than one year old. When comparing non-emergent diagnoses to intermediate and emergent diagnoses, patients 2–5 years of age, patients 6–11 years of age, and patients 12–18 years of age were less likely to use the ED for non-emergent and intermediate diagnoses (OR: 0.82, 95% CI: 0.69–0.97; OR: 0.75, 95% CI: 0.64–0.88; OR: 0.72.95% CI: 0.60–0.86, respectively) than patients less than one year of age. Females had 1.20 (95% CI: 1.08–1.31) times the odds of using the ED for less urgent diagnoses compared to males, while NHB patients had 0.87 (95% CI; 0.79–0.96) times the odds of using the ED for less urgent diagnoses compared to males. Finally, when comparing intermediate and non-emergent diagnoses to emergent diagnoses, patients with non-complex chronic and complex chronic conditions were less likely to use the ED for intermediate and non-emergent diagnoses (OR: 0.27, 95% CI: 0.24–0.30; OR 0.36, 95% CI: 0.30–0.42, respectively) than patients with non-chronic conditions. When comparing non-emergent diagnoses to emergent and intermediate diagnoses, patients with non-complex chronic and complex chronic medical complexity were less likely to use the ED for non-emergent diagnoses (OR: 0.32, 95% CI: 0.29–0.36; OR: 0.42, 95% CI: 0.36–0.49, respectively) than patients with non-chronic complexity.

**Table 2.** Multivariate Associations Between Foster Care Status and Emergency Department Utilization, Encounter-level (*n* = 14,556).

	OR, *p*-Value
No. (%)	
Foster status	
Never in foster care	Ref
Ever in foster care (E vs. I & NE)	0.87 (0.77–0.99)*
Ever in foster care (E & I vs. NE)	0.74 (0.65–0.83)*
Age at encounter	
0 year	Ref
1 year	0.82 (0.69–0.97)*
2–5 years (E vs. I & NE)	0.74 (0.63–0.87)*
2–5 years (E & I vs. NE)	0.84 (0.73–0.98)*
6–11 years (E vs. I & NE)	0.62 (0.52–0.73)*
6–11 years (E & I vs. NE)	0.75 (0.64–0.88)*
12–18 years (E vs. I & NE)	0.57 (0.47–0.69)*
12–18 years (E & I vs. NE)	0.72 (0.59–0.86)*
Sex	
Male	Ref
Female	1.20 (1.09–1.31)*
Race/Ethnicity	
Non-Hispanic white	Ref
Non-Hispanic black	0.87 (0.79–0.96)*
Hispanic	1.11 (0.76–1.61)
Other	0.89 (0.74–1.07)
Unknown	0.96 (0.57–1.61)
Medical complexity^a^	
Non-chronic	Ref
Non-complex chronic (E vs. I & NE)	0.27 (0.24–0.30)*
Non-complex chronic (E & I vs. NE)	0.32 (0.29–0.36)*
Complex chronic (E vs. I & NE)	0.36 (0.30–0.42)*
Complex chronic (E & I vs. NE)	0.42 (0.36–0.49)*

E: Emergent, I: Intermediate, NE: Non-emergent.

*Significant at .05 alpha level.

Variable categories are broken down into E versus I & NE and E & I versus NE when necessary to meet the proportional odds assumption.

^a^Defined using the Pediatric Medical Complexity Algorithm.

### Patient-Level Analysis

There were 6540 unique patients included in the analytic sample, 23.8% of whom were ever in foster care (Table 3). For patients, sex was primarily male (51.8%) and race was primarily NHW (50.3%) followed by NHB (37.5%). Hispanic patients comprised 1.8% of the patient-level sample. The initial recorded ED encounter for patients predominantly occurred between the ages of 2 and 5 years (29.7%). Non-chronic medical complexity was most common (74.8%). The majority of patients had only one ED encounter during the study period (28.0%). Patients had an average of 1.69 (Standard Deviation [SD]: 1.63, range 0–25) non-emergent ED encounters, 0.08 (SD: 0.29, range: 0–2) intermediate ED encounters, and 0.45 (SD: 0.92, range: 0–15) emergent ED encounters during the four-year study period.

**Table 3.** Descriptive and Bivariate Associations Between Demographic Characteristics and Patient-Level Necessity of ED Encounters and Foster Care Status.

	All patients (*n* = 6540)	Ever in Foster Care *N* = 1557	Never in Foster Care *n* = 4983	*p*-Value
Age at first encounter				
0 year	1050 (16.1)	364 (23.4)	686 (13.8)	<0.001
1 year	810 (12.4)	237 (15.2)	573 (11.5)	
2–5 years	1941 (29.7)	505 (32.4)	1436 (28.8)	
6–11 years	1776 (27.2)	281 (18.1)	1495 (30.0)	
12–18 years	963 (14.7)	170 (10.9)	793 (15.9)	
Sex				
Male	7541 (51.8)	816 (52.4)	2573 (51.7)	0.594
Female	7015 (48.2)	741 (47.6)	2410 (48.4)	
Race/ethnicity				
Non-Hispanic white	7347 (50.5)	731 (47.0)	2557 (51.3)	<0.001
Non-Hispanic black	5660 (38.9)	658 (42.3)	1793 (36.0)	
Hispanic	238 (1.6)	6 (0.4)	111 (2.2)	
Other	1171 (8.0)	81 (5.2)	507 (10.2)	
Unknown	140 (1.0)	81 (5.2)	15 (0.3)	
Medical complexity^a^				
Non-chronic	9275 (64.0)	1228 (78.9)	3662 (73.5)	<0.001
Non-complex chronic	3477 (24.0)	223 (14.3)	955 (19.2)	
Complex chronic	1741 (12.0)	106 (6.8)	366 (7.3)	
Time in dataset				
1 encounter	1828 (28.0)	575 (36.9)	1253 (25.2)	<0.001
Less than 1 year	1325 (20.3)	433 (27.8)	892 (17.9)	
1 year	1087 (16.6)	236 (15.2)	851 (17.1)	
2 years	1042 (15.9)	165 (10.6)	877 (17.6)	
3 years	709 (10.8)	86 (5.5)	623 (12.5)	
4 years	549 (8.4)	62 (4.0)	487 (9.8)	
Number of non-emergent ED encounters, mean (SD)^b^	1.69 (1.63)	1.3 (1.1)	1.8 (1.7)	<0.001
Number of intermediate ED encounters, mean (SD)^b^	0.08 (0.29)	0.1 (0.3)	0.7 (0.3)	<0.001
Number of emergent ED encounters, mean (SD)^b^	0.45 (0.92)	0.3 (0.8)	0.5 (1.0)	<0.001

a Defined using the Pediatric Medical Complexity Algorithm.

b Defined using the NYU-Emergency Department Algorithm.

Table 3 also displays bivariate results for the patient-level analysis. Age was significantly associated with a patient’s foster care history (*p* < .001). Patients with a history of foster care were most commonly aged between 2 to 5 years (32.4%) at their first ED encounter, while patients never in foster care were mostly 6–11 years (30.0%). Race/ethnicity was significantly associated with having a history of foster care, though those with a history of foster care and never in foster care were both most commonly NHW (47.0% and 51.3%, respectively). Both groups significantly differed by medical complexity and time in the dataset (*p* < .001 for both). Both groups most commonly had non-chronic medical complexity (36.9% history of foster care; 73.5% never in foster care) and only had one encounter to the ED (36.9% history of foster care; 25.2% never in foster care). Between patients with a history of foster care and never in foster care, number of non-emergent ED encounters (1.3 [SD: 1.1] and 1.8 [SD: 1.7], respectively), intermediate encounters (0.1 [SD: 0.3] and 0.7 [SD: 0.3]), and emergent encounters (0.3 [SD: 0.8] and 0.5 [SD: 1.0]) differed significantly (*p* < .001 for all).

Having a history of foster care was associated with a lower incidence of non-emergent encounters (Incidence Rate Ratio [IRR]: 0.83, 95% CI: 0.79–0.87, Table 4) and a higher incidence of intermediate ED encounters (IRR: 1.74, 95% CI: 1.44–2.11) compared to patients never in foster care. There was no significant difference between patients with a history of foster care and patients never in foster care in terms of incidence of emergent encounters. Patients 2–5 years of age, 6–11 years of age, and 12–18 years of age had lower incidence of non-emergent (IRR: 0.89, 95% CI: 0.84–0.94; IRR: 0.75, 95% CI: 0.70–0.80; IRR: 0.72, 95% CI: 0.66–0.77, respectively) and intermediate ED encounters (IRR: 0.65, 95% CI: 0.51–0.81; IRR: 0.44, 95% CI: 0.33–0.57; IRR: 0.25, 95% CI: 0.17–0.37, respectively) compared to patients who were less than 1 year old. Being female was associated with higher incidence of non-emergent ED encounters (IRR: 1.08, 95% CI: 1.03–1.11) but lower incidence of emergent encounters (IRR: 0.90, 0.83–0.98) than males. Being “other” race/ethnicity was associated with a lower incidence of non-emergent ED encounters (IRR: 0.89, 95% CI: 0.83–0.96) and being NHB was associated with higher incidence of emergent ED encounters (IRR: 1.17, 95% CI: 1.07–1.28) compared to NHW Having non-complex chronic medical complexity was associated with lower incidence of non-emergent ED encounters (IRR: 0.91, 95% CI: 0.86–0.96) but higher incidence of emergent ED encounters (IRR: 3.64, 95% CI: 3.30–4.01) than patients with non-chronic complexity. Finally, patients with complex chronic medical complexity had higher incidence of both non-emergent (IRR: 1.12, 95% CI: 1.04–1.20) and emergent (IRR: 3.42, 95% CI: 3.01–3.89) ED encounters compared to patients with non-chronic complexity.

**Table 4.** Incidence Rate Ratios of Emergency Department Encounter Types by Foster Care Status (*N* = 6540).

	Non-Emergent OR (95% CI)	Intermediate OR (95% CI)	Emergent OR (95% CI)
No. (%)			
Foster status			
Never in foster care	Ref	Ref	Ref
Ever in foster care	0.83 (0.79–0.87)*	1.74 (1.44–2.11)*	0.93 (0.83–1.04)
Age at first encounter			
0 year	Ref	Ref	Ref
1 year	0.97 (0.90–1.04)	0.79 (0.60–1.03)	1.13 (0.95–1.34)
2–5 years	0.89 (0.84–0.94)*	0.65 (0.51–0.81)*	0.98 (0.85–1.14)
6–11 years	0.75 (0.70–0.80)*	0.44 (0.33–0.57)*	1.01 (0.86–1.17)
12–18 years	0.72 (0.66–0.77)*	0.25 (0.17–0.37)*	1.01 (0.85–1.19)
Sex			
Male	Ref	Ref	Ref
Female	1.08 (1.03–1.11)*	1.00 (0.95–1.19)	0.90 (0.83–0.98)*
Race/Ethnicity			
Non-Hispanic white	Ref	Ref	Ref
Non-Hispanic black	1.01 (0.97–1.05)	0.89 (0.74–1.07)	1.17 (1.07–1.28)*
Hispanic	1.10 (0.95–1.29)	1.15 (0.51–2.62)	0.96 (0.69–1.33)
Other	0.89 (0.83–0.96)*	0.82 (0.58–1.16)	1.06 (0.91–1.24)
Unknown	0.99 (0.81–1.22)	1.10 (0.58–2.10)	1.21 (0.79–1.84)
Medical complexity^a^			
Non-chronic	Ref	Ref	Ref
Non-complex chronic	0.91 (0.86–0.96)*	0.85 (0.66–1.09)	3.64 (3.30–4.01)*
Complex chronic	1.12 (1.04–1.20)*	0.98 (0.70–1.39)	3.42 (3.01–3.89)*
Time in dataset			
1 encounter	Ref	Ref	Ref
Less than 1 year	1.63 (1.52–1.76)*	1.46 (1.09–1.94)*	1.46 (1.25–1.70)*
1 year	2.17 (2.02–2.34)*	1.89 (1.41–2.54)*	1.59 (1.36–1.87)*
2 years	2.70 (2.51–2.89)*	2.50 (1.86–3.35)*	2.14 (1.85–2.48)*
3 years	3.17 (2.94–3.41)*	2.46 (1.76–3.42)*	2.55 (2.18–2.98)*
4 years	4.13 (3.83–4.46)*	2.97 (2.09–4.22)*	3.47 (2.97–4.05)*

*Significant at a .017 alpha level.

^a^Defined using the Pediatric Medical Complexity Algorithm.

## Discussion

This study described preventable ED utilization of patients with foster care history seen at a large, urban, pediatric ED and compared it to utilization by patients without foster care history using the Andersen behavioral model of health care services use as a theoretical framework. Contrary to our hypothesis that encounters with patients with foster care experience would be more likely to be preventable than encounters with patients without foster care experience, we found that ED encounters with patients with a history of foster care were less likely to be preventable. Further, and also contrary to our hypothesis, when exploring data at the individual patient level, we found that patients with foster care history were less likely to use the ED for concerns that did not need immediate attention but were more likely to use the ED for intermediate reasons.

One possible explanation for a lower probability of patients with foster care experience using the ED for preventable reasons may be the quality of primary care received, which would serve as an enabling factor, according to the Andersen model. The hospital system has an integrated primary care clinic that exclusively serves patients with child welfare involvement. This specialized, “one-stop shop” form of primary care may be decreasing the likelihood of patients utilizing the ED for preventable reasons. The Rees-Jones Center for Foster Care Excellence was the first clinic to operationalize the AAP guidelines for primary healthcare for children in foster care, which include seeing children early and often, delivering comprehensive and trauma-informed integrated behavioral health services, and providing care coordination between healthcare providers and child welfare personnel (Task Force on Health Care for Children in Foster Care, 2005). They also provide caregiver education and support and healthcare support during the transition into and out of foster care.

Further, in Texas, foster caregivers receive support services from Child Protective Services and other child welfare agencies, have requirements such as being financially stable and completing initial and ongoing training related to health care and navigating resources, and are required to take a child for a medical exam within three days of coming into care and a full checkup within 30 days of coming into care (Texas Department of Family and Protective Services, n.d). These factors may help to decrease preventable ED encounters. Indeed, research shows that despite established relationships with primary care providers and positive perceptions of primary care services, many caregivers of children still utilize the ED for financial reasons or lack of awareness of primary care resources (Kirby et al., 2021; Ravi et al., 2021). Further research is needed to determine if these findings hold in a population of patients in foster care who do not have access to this type of specialized primary care.

It is also important to note that ED utilization for mental health reasons is increasing rapidly, with encounters for these reasons increasing by 8% annually between 2011 and 2020, yet many ED encounters for mental health reasons are avoidable (Bommersbach et al., 2023; Lindsey, 2022). Mental health status qualifies as a need factor in the Andersen model. Children in foster care have higher proportions of mental and behavioral health conditions and may be more likely than children not in foster care to use the ED for these reasons. Another study showed that of children in foster care, those with personality disorders were 9.4 times as likely to use the ED, and those with depression were 5.4 times as likely to use the ED than those without these conditions (Almgren & Marcenko, 2001). In the current study, ED encounters for mental health reasons were not included because the NYU-ED algorithm classifies the ICD-10 F codes (i.e., codes indicated mental, behavioral and neurodevelopmental disorders) into “psych”, “alcohol”, and “drug” categories, rather than classifying their urgency, which may have impacted these results. To truly understand the ED utilization of patients in foster care, it will be important to incorporate psychiatric conditions into assessments of ED necessity rather than its own category and to explore these results in a group of patients who do not receive integrated behavioral health services.

The findings that patients with a history of foster care had a higher incidence of ED encounters for intermediate reasons and the same incidence of ED visits for emergent encounters as patients never in foster care are not too surprising. The two categories that comprise intermediate encounters are Emergent/Primary Care Treatable and Emergent- ED Care Needed- Preventable/Avoidable. To classify an encounter as Emergent-ED Care Needed- Preventable/Avoidable, the diagnosis is required to be preventable with maintenance care from a primary care provider. Possible explanations for the higher incidence of utilization for intermediate reasons for patients with a history of foster care include that (a) the patient’s health was not maintained or properly treated prior to coming into care (which is a reason for a child being removed from a biological parent) such that health issues have escalated to needing ED intervention by the time the child comes into care and (b) foster parents may lack the full health history of a child in their care and thus err on the side of caution when a health concern arises. In one qualitative study, foster caregivers identified receiving little to no medical history information as a major challenge, impacting their ability to maintain previously established healthcare resources such as prescriptions (Greiner et al., 2015). Further, having a usual source of health care has been identified as an enabling factor in the Andersen model, and a disruption of this usual source or lack of such a source could potentially increase the perceived need of ED services (Babitsch et al., 2012). The finding warrants greater attention on the part of Child Protective Services and health care providers to obtaining past health-related records on a child as quickly as possible and to communicating the information contained in those records to foster parents. The finding also suggests that health care providers should spend time discussing symptoms associated with a particular diagnosis and what might warrant an ED encounter versus what could be treated in the primary care setting, along with appropriate preventive and chronic care.

Several demographic/predisposing factors were associated with ED utilization. Being older was associated with a lower probability of using the ED for less urgent concerns and lower incidence of using the ED for non-emergent and intermediate diagnoses. This finding is consistent with systematic reviews finding that children under the age of 5 are more frequent ED utilizers and that younger age is a predictor of non-urgent ED use (Alele et al., 2019; Greenfield et al., 2021). Reasons for higher ED utilization among young patients likely include the increased need for reassurance among parents, inexperience with parenting, and increased communication challenges with infants and toddlers (Butun et al., 2018; Hendry, Beattie, & Heaney, 2005).

Being female was associated with a higher probability of using the ED for less urgent complaints, a higher incidence of non-emergent ED encounters, and a lower incidence of emergent ED encounters. Past research has conflicting results on whether male or female children used the ED more frequently for non-urgent reasons, with a systematic review finding that of 3 studies, 2 demonstrate females having higher non-urgent ED utilization and one demonstrating lower ED utilization (Alele et al., 2019). These results add to the argument that sex should be further explored when assessing non-urgent ED utilization.

Finally, NHB children were less likely to use the ED for less urgent complaints and had more emergent ED encounters, while being other race was associated with lower incidence of non-emergent ED encounters. This is inconsistent with a nationally representative study of ED data demonstrating that NHB children were less likely to use the ED for emergent encounters than NHW children. However, this study did demonstrate higher mortality rates among NHB children, indicating dissonance between risk assessment and outcomes (Zhang et al., 2019). This study used the Emergency Severity Index (ESI) rather than the NYU-ED algorithm to determine emergent encounters. ESI is assigned upon presentation to the ED and is used as a triage tool. It takes four criteria (i.e., 1. Condition requires lifesaving intervention; 2. High-risk situations [e.g., altered mental status, severe pain]; 3. The number of resources typically needed for similar conditions; and 4. If the patient has high-risk vital signs) into account and classifies each patient’s risk on a scale from 5 to 1 with 5 being highest acuity (Emergency Nurses Association, n.d). However, differences in ESI scores between NHB and NHW pediatric patients have been demonstrated that are unexplained by clinical and utilization characteristics, with NHB patients having 1.89 times the odds (95% CI; 1.69–2.12) of lower acuity scores than NHW patients indicating the potential presence of racial disparities and systematic bias in healthcare treatment (Zook et al., 2016). The NYU-ED algorithm uses the discharge diagnosis code determined by the physician post-treatment, and may be more accurate in determining the true urgency of ED encounters. These results demonstrate that NHB patients are presenting to the ED with emergent reasons, support the idea that NHB patients may be triaged at lower severity than their true need, and that ESI may not accurately reflect the severity of ED encounters. The reason that NHB children ultimately use the ED for more emergent reasons in the first place is likely related to the poorer quality health care that they receive in a multitude of medical settings than NHW children (Slopen et al., 2024). Receiving poorer health care can lead to exacerbation of acute and chronic medical conditions, ultimately requiring emergency treatment. These findings highlight the importance of incorporating implicit bias and cultural awareness training and intervention into care teams in both the ED and other settings. Further, intervention to improve health care for minoritized children in settings that precede the ED may help improve the social determinants of health drivers for the disparity in ED urgency. The decreased rate of non-urgent encounters among “other” race patients is difficult to interpret due to the combination of various races and different races included in different studies.

Our study found that having a non-complex chronic or complex chronic medical condition was associated with lower odds of using the ED for less urgent reasons which is consistent with the Andersen model theory that health care need is associated with higher health care utilization. Further, patients with non-complex chronic conditions had a lower incidence of non-emergent encounters but higher incidence of emergent encounters than those with non-chronic conditions. Patients with complex chronic conditions had a higher incidence of both non-emergent and emergent encounters compared to those with non-chronic conditions. In this study, there was a larger proportion (36.0%) of ED encounters among patients with chronic medical conditions than in other published literature, demonstrating a range of 1.8–24.0% of ED encounters for children with such conditions (O’Mahony et al., 2013). O’Mahony et al. also demonstrated that increasing medical complexity is associated with an increased length of stay (79 minutes, 95% CI: 77–81) and subsequent hospital admission (hospital OR: 10.3, 95% CI: 9.9–10.7; Pediatric Intensive Care Unit (OR: 25.0, 95% CI: 17.0–36.0), consistent with our results that patients with these conditions use the ED for more urgent reasons (O’Mahony et al., 2013). Interestingly, the proportion of patients with non-complex chronic and complex chronic medical conditions was similar among patients with and without a history of foster care in this sample, which is not consistent with that the designation of children in foster care as children with special healthcare needs opposed to the general population (Council on Foster Care et al., 2015). This may be related to the fact that both patients with and without a history of foster care access primary care services affiliated with a hospital system, an indication they may need more specialized care and access to services. Because these patients have access to these services through the primary care clinic, this may contribute to the decrease in less emergent ED use. To our knowledge, this study is the first to specifically explore the necessity of encounters between chronicity and complexity types.

There are several limitations to this study. First, we were unable to exclusively explore patients who were currently in foster care, as the de-identified nature of the data set meant that we could only discern patients who had ever been in foster care during the study period. Secondly, our sample included only patients seen at either the Rees-Jones Center for Foster Care Excellence or a primary care clinic at the hospital associated with the ED of study. Both of these factors limit our generalizability to patients with a history of foster care receiving primary care at one academic pediatric health system. It does, however, permit us to compare children who have access to primary care. Thirdly, we could not include important covariates related to healthcare utilization, such as urbanicity, due to data availability and insurance because all patients with a history of foster care are offered Star Health which is not available to patients never in foster care. This may have impacted our effect estimates and also limited our ability to use enabling factors from the Andersen model as covariates. Fourth, because of the structure of the NYU-ED algorithm, we were unable to explore the necessity of ED encounters related to mental and behavioral health. Finally, patients seen at the Rees-Jones Center for Foster Care Excellence or who had Star Health insurance during at least one ED encounter were flagged as having a history of foster care. There is the potential that patients with a history of foster care were classified as never in foster care if they were ever in foster care but were not seen at the Rees-Jones Center for Foster Care Excellence.

This study demonstrated that patients with a history of foster care were less likely to use the ED for non-urgent reasons but more likely to use the ED for intermediate reasons compared to patients never in foster care. This finding suggests that these patients may be a poor target for intervention planning to decrease non-urgent ED encounters and spending in the United States. However, interventions targeted to improving provision of health care services to patients in foster care upon entry to the foster care system and during transitions may improve preventable ED utilization for intermediate reasons among this group. Future research is needed to determine if these results are consistent in other foster care populations. Results also demonstrate potential target intervention groups with higher non-emergent ED utilization including younger patients, female patients, and patients without chronic healthcare conditions.
